# DBtRend: A Web-Server of tRNA Expression Profiles from Small RNA Sequencing Data in Humans

**DOI:** 10.3390/genes12101576

**Published:** 2021-10-03

**Authors:** Jin-Ok Lee, Minho Lee, Yeun-Jun Chung

**Affiliations:** 1 Department of Biomedicine & Health Sciences, Graduate School, The Catholic University of Korea, Seoul 06591, Korea; jinoklee@catholic.ac.kr; 2 Department of Life Science, Dongguk University-Seoul, Goyang 10326, Korea; 3 Department of Microbiology, College of Medicine, The Catholic University of Korea, Seoul 06591, Korea; 4 Precision Medicine Research Center, College of Medicine, The Catholic University of Korea, Seoul 06591, Korea; 5 Integrated Research Center for Genome Polymorphism, College of Medicine, The Catholic University of Korea, Seoul 06591, Korea

**Keywords:** human tRNA expression, small RNA sequencing, web database, web-based interactive analysis

## Abstract

Transfer RNA (tRNA), a key component of the translation machinery, plays critical roles in stress conditions and various diseases. While knowledge regarding the importance of tRNA function is increasing, its biological roles are still not well understood. There is currently no comprehensive database or web server providing the expression landscape of tRNAs across a variety of human tissues and diseases. Here, we constructed a user-friendly and interactive database, DBtRend, which provides a profile of mature tRNA expression across various biological conditions by reanalyzing the small RNA or microRNA sequencing data from the Cancer Genome Atlas (TCGA) and NCBI’s Gene Expression Omnibus (GEO) in humans. Users can explore not only the expression values of mature individual tRNAs in the human genome, but also those of isodecoders and isoacceptors based on our specific pipelines. DBtRend provides the expressed patterns of tRNAs, the differentially expressed tRNAs in different biological conditions, and the information of samples or patients, tissue types, and molecular subtype of cancers. The database is expected to help researchers interested in functional discoveries of tRNAs.

## 1. Introduction

Transfer RNAs (tRNAs) are key components of the translation machinery that function as adaptors to deliver amino acids to the ribosome by matching mRNA codons with their corresponding amino acids. The abundance of tRNAs varies across biological conditions including tissue type [1], cell cycle [2], developmental stage [3], and disease status (e.g., diabetes mellitus [4], Huntington’s disease [5], and cancers [6]). These studies highlight the need to comprehensively and systemically identify how tRNA expression differs in tissues and biological conditions. Although several studies have identified tRNA expression profiles, the field is currently limited by the small number of samples and studies and the lack of exploration of various biological conditions. One reason for the insufficient number of studies related to tRNA expression profiles is the technical difficulty in quantifying individual tRNAs caused by post-transcriptional modification and formation of tRNA structures. The indirect quantification of tRNA expression from small RNA has been proposed as one approach to overcome these limitations. Small RNA sequencing data presents limitations in the quantification accuracy of mature tRNA due to the quantification bias which caused the complexity of the tRNA structure and difficulties in discriminating reads from premature tRNAs, mature tRNAs, or tRNA-derived fragments (tRFs). Although tRNA sequencing and pipelines are being developed to overcome these limitations, they are still insufficient. Despite these limitations, tRNA expression in small RNA sequencing datasets has been studied [7,8,9,10,11]. In addition, the Cancer Genome Atlas (TCGA) has been used to investigate alterations in tRNA expression in cancers [12,13]. However, there is a lack of studies on a variety of biological conditions.

Depositing datasets in the Gene Expression Omnibus (GEO) of the National Center for Biotechnology Information (NCBI) ensures the reproducibility of published studies and promotes their reuse. Large consortium projects, such as TCGA, have provided opportunities to systematically characterize molecular mechanisms. Several re-analysis studies on these data provided access to the integration of large datasets to simplify their use and supported functionalities for downstream analyses (such as exploratory data analysis, differential expression analysis, and survival analysis). For example, cBioPotal is an online resource for exploring, visualizing, and analyzing multidimensional cancer genomic datasets and provides rapid and intuitive access to molecular profiles and clinical properties from large consortium projects [14]. Gene Expression Profiling Interactive Analysis (GEPIA) is a web server that provides interactive and customizable functions of gene expression patterns using datasets from TCGA and Genotype-Tissue Expression (GTEx) [15]. The database of differentially expressed miRNAs in human cancers (dbDEMC) is a web database using datasets from GEO, TCGA, and the International Cancer Genome Consortium (ICGC) [16]. The Gene Expression Atlas is a database that provides information about gene and protein expression in different studies and organisms, including plants and metazoans, based on GEO, the European Nucleotide Archive (ENA), and ArrayExpress data [17]. These resources present tools for researchers to obtain novel scientific insights, and they have been used to inform the design of new studies. Although there are various resource studies, these resources are usually used for mRNA or miRNA analysis.

**Figure 1:**
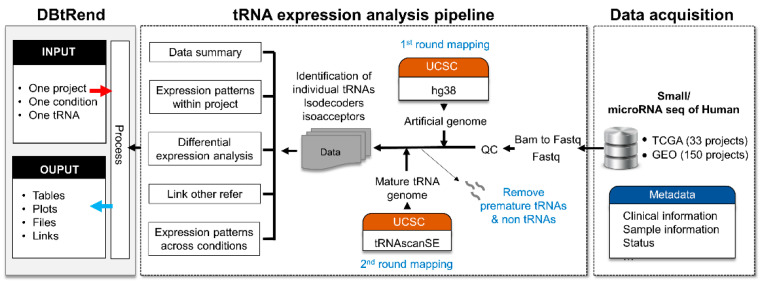
Schematic representation of the data processing and workflow of DBtRend construction (https://trend.pmrc.re.kr/, accessed on 10 June 2020). The DBtRend re-analyze human small RNA sequencing datasets such as microRNA sequencing datasets from GEO and TCGA. We then calculated the expression of mature tRNAs using a specific pipeline and merged them into the isodecoder and isoacceptor levels.

For tRNA expression, OncotRF is an online resource for query and visual exploration of tRFs using small RNA sequencing data from TCGA [18]. A recently developed web resource, tRic, is unique in its exploration of the expression landscape of tRNAs in human cancers based on TCGA [19]. Here, we present a more comprehensive web resource named DBtRend (DataBase of tRNA expressions of various conditions including normal and diseased samples, https://trend.pmrc.re.kr/ accessed on 10 June 2020), which provides and utilizes standardized tRNA expression profiles in different tissues, cell types, developmental stages, and other conditions, including cancer, from public datasets (TCGA and GEO) (Figure 1). This resource enables users to find biological conditions or tissue types where a specific tRNA expression level is differentially expressed. In addition, users can retrieve differentially expressed tRNAs depending on their specific biological condition or disease state.

## 2. Materials and Methods

### 2.1. Data Collection

We downloaded microRNA sequencing from the TCGA portal (https://portal.gdc.cancer.gov/, accessed on 15 January 2020) approved by the dbGaP (dbGaP accession: phs000178.v11.p8). To collect the small RNA sequencing data from GEO (https://www.ncbi.nlm.nih.gov/geo/, accessed on 10 June 2020), project IDs were first obtained by searching “Non-coding RNA profiling by high throughput sequencing” for each experiment type on two platforms: GPL10999 (Illumina Genome Analyzer IIx [Homo sapiens]) and GPL11154 (Illumina HiSeq 2000 [Homo sapiens]). We subsequently used the GEOmetadb R package [20], and metadata of the searched project were obtained using the GEOmetadb R package [20]. Human samples annotated with small RNA sequencing and micro RNA sequencing for each metadata in the project were downloaded using SRA tool kits (https://trace.ncbi.nlm.nih.gov/Traces/sra/sra.cgi?view=software, accessed on 16 August 2020). The sample information was obtained from the website, and molecular subtype information of TCGA was obtained from TCGAbiolinks [21]. Information on the tRNA gene id was obtained from GtRNAdb [22].

### 2.2. tRNA Identification

Recently, we applied a novel quantification method that modified previous methods [11,12] for tRNA genes and developed a pipeline in the R programming environment (https://github.com/jinoklee/tReasure, accessed on 4 August 2020) from raw sequencing data (FASTQ files). To apply this method, BAM files from the TCGA data portal were converted into FASTQ using SAMtools [23]. Raw FASTQ files were pre-processed with adaptor removal and quality filtering by applying Cutadapt (-m 10-M 50-q 25) [24]. Trimmed sequences were mapped using a modified specific pipeline for mature tRNA [11]. Detailed methods are described in https://github.com/jinoklee/tReasure/blob/master/doc/Detailed-Mapping-Methods.pdf (accessed on 24 September 2021). In brief, we built two genomes for two mapping steps, an artificial human genome and a human mature tRNA genome, using predicted tRNA sequences. Human tRNA gene annotations (hg38) were obtained using tRNAscan-SE [25], which predicted all tRNA sequences (cytosolic and mitochondrial tRNAs) and provided a score assigned to each putative tRNA gene. tRNAs with high confidence scores were considered to be those most likely to function in translation by assessing a combination of domain-specific, isotype-specific, and secondary structure scores. Preprocessed reads were first mapped against the artificial human genome, masking all annotated tRNAs and adding premature tRNAs as extra chromosomes. After the first round mapping step, the reads that mapped to the tRNA-masked genomic region and premature tRNA region were removed using a customized R code. Filtered reads were mapped against the mature tRNA genome, in which tRNAs were generated by appending 3′-CCA tails and removing introns to the predicted tRNA sequence. For reliable and accurate quantification of mature tRNAs, we selected tRNAs with high confidence values rather than the whole set of predicted mature tRNAs. Only cytosolic tRNAs were selected for further analyses. The number of reads mapped to individual tRNAs was counted using SAMtools [23]. These tRNAs were categorized into 429 individual tRNAs, 259 isodecoders, and 48 isoacceptors (including two initiator tRNAs, iMet-tRNAiMet). Raw individual tRNA counts were merged into matched isoacceptors and isodecoders. Prior to downstream analysis, tRNA expression was normalized as the log2 counts per million (CPM) and then adjusted for potential batch effects with sequencing plates (TCGA) or the batch information (GEO) as a covariate using ComBat [26]. DBtRend additionally provided the expression matrix of the individual tRNA normalized as reads per kilobase of transcript per million mapped reads (RPKM) and transcripts per million (TPM).

### 2.3. Differential Expression Analysis

All statistical tests were performed using R. To compare the expression profile patterns of tRNAs across biological conditions, we utilized projects that included at least two different conditions and biological replicates. We used the edgeR package to examine the differential expression of groups using the trimmed mean of M values (TMM) by normalized methods. tRNAs with an average log2 CPM < 1 were removed. Quasi-likelihood F-tests were used for testing, and the Benjamini-Hochberg (BH) procedure was applied for multiple corrections.

## 3. Results

### 3.1. Data Statistics in DBtRend

**Figure 2:**
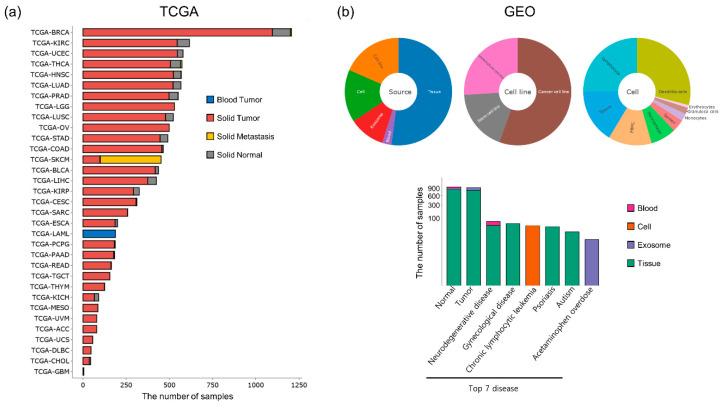
Summary of the data statistics in DBtRend. (**a**) The number of samples in different TCGA cancer types. (**b**) The numbers of samples in different source and top 7 disease from GEO datasets.

Currently, the DBtRend is based on 183 projects, investigating 33 cancers from TCGA and 150 projects from GEO. As a result of quality control of the sequencing raw data, DBtRend provided 14,986 out of 15,170 samples, comprising 11,078 samples from TCGA and 3908 samples from GEO, sequenced using GPL11154 (Illumina HiSeq 2000: 3077 samples), GPL10999 (Illumina Genome Analyzer IIx: 770 samples), GPL9115 (Illumina Analyzer II: 22 samples), and GPL15520 (Illumina MiSeq: 39 samples). Figure 2 shows the distribution of the samples in DBtRend.

For differentially expressed tRNA gene (DEtRNA) analysis, we selected 105 of 183 projects, comprising 21 projects from TCGA with at least 2 samples of matched normal and tumor samples, and 84 projects from GEO with 2 replicates per condition. These results are displayed on the ‘Explore DEtRNA’ page.

### 3.2. Database Infrastructure

The DBtRend was constructed based on the R programming language (version 4.0) using the ‘shiny’ web application framework package. The web interface is based on the package ‘semantic.dashboard’, HTML and CSS. We will continue to maintain a database for possible updates. Based on these datasets, DBtRend provide four main modules to retrieve various data: ‘Home’, ‘Explore Projects’, ‘Explore DEtRNAs’ and ‘Explore tRNAs’. A detailed tutorial concerning the exploration of the DBtRend is available on the ‘User Guide’ page.

#### 3.2.1. ‘Home’ Page

The sample distribution is shown on the ‘Home’ page. Four pie charts display the sample percentage of ‘project’, ‘source’, ‘cell line’, and ‘cell’ parts, respectively. A table with detailed project information is displayed on this page. The table shows ‘Project’, ‘Study.title’, ‘Source’, ‘Instrument’, ‘Sample.n’, and ‘Reference’. By clicking the name on the pie chart, the table is reconstructed to display the project of the matched samples.

#### 3.2.2. Query on the ‘Explore Projects’ Page

**Figure 3:**
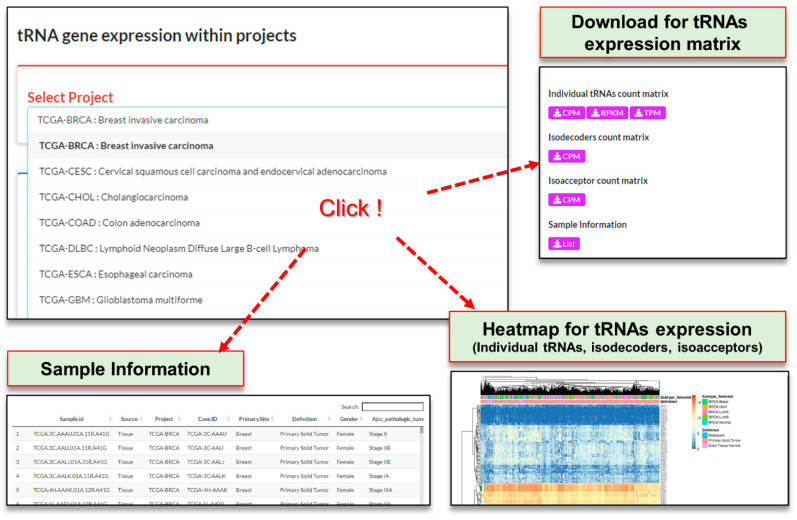
The ‘Explore Projects’ page.

Users can perform a search by selecting a project from a pull-down menu. The query results are displayed as a table of sample information in the tab of ‘Sample Information’ and three heatmaps, visualizing the tRNAs expression profiles within the project in each corresponding tab (‘Individual tRNAs’, ‘Isodecoders’ and ‘Isoacceptors’). In the sample list, ‘Sample.id’, ‘Source’, ‘Project’ and ‘DE.Group’ (group information given when analyzing tRNA gene differential expression) are commonly provided for each project. There may be additional content depending on the project. Heatmaps also provide hierarchical clustering of samples based on the relative abundance of all tRNA genes. DBtRend provides two types of static and interactive heatmaps using the pheatmap R package and heatmaply R package. The names of the tRNAs can be displayed by positioning the cursor over each of the points in the interactive heatmap. Using the sub-tab ‘Download’, users can download normalized read counts as the matrix of CPM (counts per million) and the list of sample information (Figure 3). In addition, matrices of RPKM and TPM were provided for individual tRNAs.

#### 3.2.3. Query on the ‘Explore DEtRNAs’ Page

For the analysis of DEtRNAs within that project, users should search a project by clicking the pull-down menu and then selecting a comparison in the project from the pull-down menu shown below. To search for statistically significant results, users can select the threshold values of logFC and the adjusted p-value. The results are displayed in tables of statistical tests and a plot of the results of DEtRNAs. A results table with ‘GeneName’, ‘logFC’, ‘logCPM’, ‘F’, ‘PValue’, ‘FDR’ and ‘Sig’ is displayed in each corresponding tabs.

**Figure 4:**
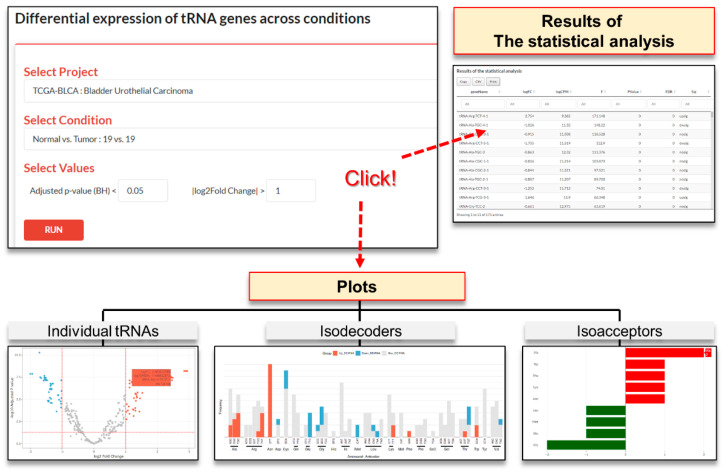
The ‘Explore DEtRNAs’ page.

Three plots will be displayed: a volcano plot for individual tRNAs, a bar plot for isodecoders, and a pyramid plot for isoacceptors in each corresponding table. By positioning the cursor over each of the points in the volcano plot, the names of the tRNAs are displayed (Figure 4).

#### 3.2.4. Query on the ‘Explore tRNAs’ Page

**Figure 5:**
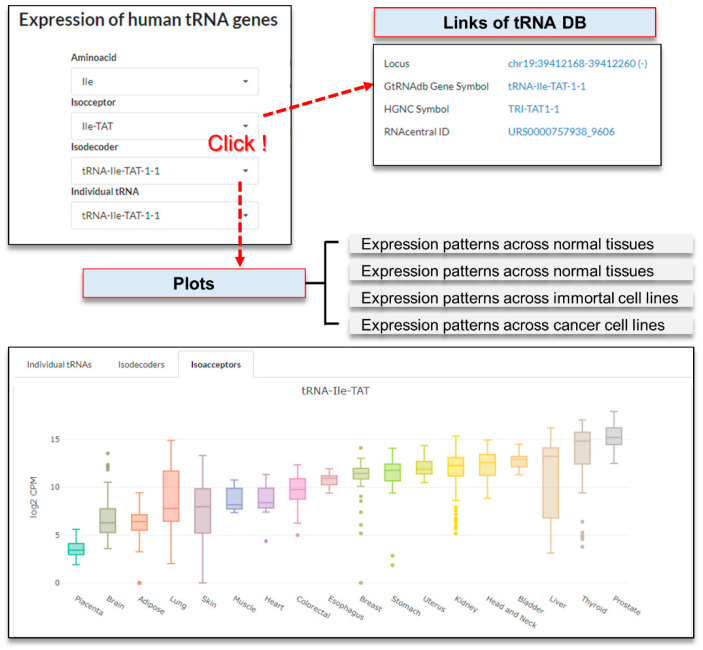
The ‘Explore tRNAs’ page.

To query tRNAs, individual tRNAs must be selected step-by-step from a pull-down menu. The query results will display four links to other databases for the tRNA, including UCSC Genome Browser (http://genome.ucsc.edu/, accessed on 5 September 2021), GtRNAdb (http://gtrnadb.ucsc.edu/genomes/eukaryota/Hsapi38/, accessed on 5 September 2021), HGNC (https://www.genenames.org/, accessed on 5 September 2021), and RNAcentral (https://rnacentral.org/, accessed on 5 September 2021), on the right side. Users can find a specific reference page for the matched tRNAs by clicking on the link text. In addition, a boxplot was displayed to represent the expression pattern across 18 normal tissues, 24 tumor tissues, 21 immortal cell lines, 37 cancer cell lines, and groups in a selected project for the selected individual tRNAs, isodecoders, and isoacceptors, respectively. By positioning the cursor over each box plot, the summarized statistical values (‘minimum’, ‘first quartile’, ‘median’, ‘third quartile’, and ‘maximum’) will be shown (Figure 5).

#### 3.2.5. An Example Use: Alzheimer’s Disease

To search for the Alzheimer’s disease (AD) project in DBtRend, we typed ‘Alzheimer’ in the search box of the table directly on the ’Home’ page. The results were displayed in three GEO projects with blood or tissue sources. To explore tRNA gene expression, one project (PRJNA210613) was explored by selecting the pull-down on the Explore Project page. We selected the project, ‘PRJNA210613: Alternation of the microRNA network during the progression of Alzheimer’s disease’. The query results are presented below. The ‘Sample Information’ tab displayed six samples each from prefrontal cortex between the early and the late stage group of AD groups. Heatmaps for the expression pattern of tRNA genes were displayed by clicking the subsequent three tabs. Each heat-map-based hierarchical clustering of samples separated early and late stages of AD for DEtRNA expression within projects; then, we went to the ‘Explore DEtRNAs’ page. First, we searched for the project and then verified the comparison condition (such as early stage vs. late stage: 6 vs. 6′) in the following filter. DBtRend displayed DEtRNAs in the late-stage group compared with the early stage group. We identified 118 individual tRNAs (61 upregulated and 57 downregulated individual tRNAs), 72 isodecoders (29 upregulated and 43 downregulated isodecoders), and 14 isoacceptors (six upregulated and eight downregulated isoacceptors) in the late stage of AD compared with the early stage. From the results of this analysis, two interesting results were identified. First, the most discovered the differential expressed isodecoders were encoding cysteine, followed by arginine on bar plot in the ‘isodecoder’ Table These isodecoders could be proposed as novel critical factors related to AD. Second, the isodecoders and isoacceptors transferring arginine were up- or down-regulated between the conditions. This suggests that each tRNA encoding arginine with different anticodons has a different function. This needs to be further studied and suggested as a candidate marker for follow-up work.

## 4. Discussion

DBtRend is an interactive web-based database to explore and investigate tRNA expression under various biological conditions retrieved from the TCGA and GEO datasets. This data collection was larger than that of previous studies using TCGA data [19]. We will continuously collect and analyze not only small RNA sequencing data, but also tRNA sequencing data. Currently, DBtRend excludes premature tRNAs using special pipelines, but there is a limitation in that it is difficult to distinguish expression levels of tRFs from those of mature tRNAs in small RNA sequencing data. This issue will be addressed in a subsequent development algorithm that quantifies tRNA expressions more precisely.

## 5. Conclusions

We developed a comprehensive database, DBtRend, for tRNA expression under various biological conditions. We provide the tRNA expression profile along with differential expression across biological conditions and among different groups of samples at individual tRNA, isodecoder, and isoacceptor levels. DBtRend will be useful for studying human biology and applied medical research, such as biomarker discovery.

## Data Availability

The datasets produced in this study are available in the DBtRend (https://trend.pmrc.re.kr/).
